# Effect of Water Microsolvation on the Excited-State Proton Transfer of 3-Hydroxyflavone Enclosed in γ-Cyclodextrin

**DOI:** 10.3390/molecules26040843

**Published:** 2021-02-05

**Authors:** Khanittha Kerdpol, Rathawat Daengngern, Chanchai Sattayanon, Supawadee Namuangruk, Thanyada Rungrotmongkol, Peter Wolschann, Nawee Kungwan, Supot Hannongbua

**Affiliations:** 1 Center of Excellence in Computational Chemistry (CECC), Department of Chemistry, Faculty of Science, Chulalongkorn University, Bangkok 10330, Thailand; khanittha.view@gmail.com; 2 Department of Chemistry, Faculty of Science, King Mongkut’s Institute of Technology Ladkrabang, Bangkok 10520, Thailand; rathawat.da@kmitl.ac.th; 3 Integrated Applied Chemistry Research Unit, Faculty of Science, King Mongkut’s Institute of Technology Ladkrabang, Bangkok 10520, Thailand; 4 National Nanotechnology Center (NANOTEC), NSTDA, 111 Thailand Science Park, Pahonyothin Road, Klong Luang, Pathum Thani 12120, Thailand; c.sattayanon@gmail.com (C.S.); supawadee@nanotec.or.th (S.N.); 5 Structural and Computational Biology Research Unit, Department of Biochemistry, Faculty of Science, Chulalongkorn University, Bangkok 10330, Thailand; t.rungrotmongkol@gmail.com; 6 Program in Bioinformatics and Computational Biology, Graduate School, Chulalongkorn University, Bangkok 10330, Thailand; 7 Molecular Sensory Science Center, Faculty of Science, Chulalongkorn University, Bangkok 10330, Thailand; 8 Department of Pharmaceutical Chemistry, University of Vienna, 1090 Vienna, Austria; karl.peter.wolschann@univie.ac.at; 9 Institute of Theoretical Chemistry, University of Vienna, 1090 Vienna, Austria; 10 Department of Chemistry, Faculty of Science, Chiang Mai University, Chiang Mai 50200, Thailand; 11 Center of Excellence in Materials Science and Technology, Chiang Mai University, Chiang Mai 50200, Thailand

**Keywords:** 3-hydroxyflavone (3HF), γ-cyclodextrin (γ-CD), excited-state proton transfer (ESPT), molecular dynamics (MD), density functional theory (DFT)

## Abstract

The effect of microsolvation on excited-state proton transfer (ESPT) reaction of 3-hydroxyflavone (3HF) and its inclusion complex with γ-cyclodextrin (γ-CD) was studied using computational approaches. From molecular dynamics simulations, two possible inclusion complexes formed by the chromone ring (C-ring, Form I) and the phenyl ring (P-ring, Form II) of 3HF insertion to γ-CD were observed. Form II is likely more stable because of lower fluctuation of 3HF inside the hydrophobic cavity and lower water accessibility to the encapsulated 3HF. Next, the conformation analysis of these models in the ground (S_0_) and the first excited (S_1_) states was carried out by density functional theory (DFT) and time-dependent DFT (TD-DFT) calculations, respectively, to reveal the photophysical properties of 3HF influenced by the γ-CD. The results show that the intermolecular hydrogen bonding (interHB) between 3HF and γ-CD, and intramolecular hydrogen bonding (intraHB) within 3HF are strengthened in the S_1_ state confirmed by the shorter interHB and intraHB distances and the red-shift of O–H vibrational modes involving in the ESPT process. The simulated absorption and emission spectra are in good agreement with the experimental data. Significantly, in the S_1_ state, the keto form of 3HF is stabilized by γ-CD, explaining the increased quantum yield of keto emission of 3HF when complexing with γ-CD in the experiment. In the other word, ESPT of 3HF is more favorable in the γ-CD hydrophobic cavity than in aqueous solution.

## 1. Introduction

Fluorescent organic molecules with strong intramolecular hydrogen bonds (intraHBs) connected by proton donating and accepting groups have gained considerable attention in recent years owing to their unique fluorescent emission in term of large Stokes shift without self-absorption [1]. Their unique properties harnessing from the excited state intramolecular proton transfer (ESIntraPT) could be typically described by the characteristic four-level photocycle. Initially, the molecule in an enol form (E) in the ground (S_0_) state absorbing light in the shorter wavelength region results in the photoexcitation process from the S_0_ state into the excited (S_1_) state. The intraHB of E is strengthened in the S_1_ state because of the charge redistribution upon photoexcitation, leading to the transfer of proton from the donor (D: −NH_2_, −OH) to the acceptor (A: C=O, −N=), which changes the enol form (E*) to the keto form (K*) in the S_1_ state. After that, the K* emits the fluorescence in the remarkably longer wavelength than the absorption and relaxes to the S_0_ state, resulting in the notably large Stokes shift (the difference between positions of absorption and emission peaks). Then the K changes to the E through the back proton transfer (BPT) process spontaneously in the S_0_ state due to the PT barrierless and high exothermic reaction. Generally, their photophysical properties can be easily modulated using many strategies [2,3] such as introducing electron-donating and withdrawing substituents, the heteroatom substitution, and the π-conjugation into the main core structure, to give the desirable absorption and emission spectra as well as large Stokes shift. The ESIntraPT molecules with such tunable photophysical properties including derivatives of salicylates [4,5,6], salicylideneanilines [7,8,9], flavones [10,11,12,13], benzazoles [14,15,16,17,18], and chalcones [19,20,21] have been reported and widely used in various applications ranging from chemical sensing to light-emitting diodes [22,23,24,25,26,27].

Among various ESIntraPT molecules, 3-hydroxyflavone (3HF), which consists of a chromone ring (C-ring) and a phenyl ring (P-ring), is one of the best-known molecular systems. 3HF exhibits ESIntraPT and gives dual fluorescence corresponding to its E* and K* forms with a large Stokes shift and photostability [28,29,30]. Thus, 3HF has been used as a prototype for the ESIntraPT processes and as sensitive fluorescence probes for discovering binding sites in various bio-relevant targets such as DNA, protein, and bio-membranes [31,32,33]. The photophysical properties and ESPT processes of 3HF in organic solvents have been extensively studied [34,35,36,37,38,39,40,41,42]. In non-polar solvent, only the K* emission peak of 3HF in toluene was observed at 530 nm with large Stokes shift [33] because the ESIntraPT process effectively occurs, giving only K* form. However, in a protic solvent, the dual emission peaks from E* and K* of 3HF in methanol were observed at 409 and 528 nm, respectively [33], because the IntraHB of 3HF is disrupted and the intermolecular hydrogen bonds (interHBs) between 3HF and protic solvents is formed depending on the nature of solvents and the arrangement of protic solvent around 3HF. This favorable formation of interHBs could reduce the formation of K*, resulting in the low quantum yield in protic solvents or aqueous solution [40,41,42,43,44].

One of the strategies to enhance the K* emission intensity of 3HF in an aqueous solution is disrupting the interHBs of 3HF-water cage-like network using cyclodextrin (CD) [45,46,47]. The reduction of polarity and restricted environment inside CD’s cavity is essential for many aspects of photophysical phenomena by inclusion complexes between 3HF and CD [45,46,47]. CDs are the cyclic oligosaccharides consisting of the crucial α-d-glucose unit, which exhibit the conical shape and the α-d-glucose units of 6, 7, and 8 are represented as α-, β-, and γ-cyclodextrins (α-, β-, and γ-CDs), respectively [48]. S. Das and N. Chattopadhyay experimentally studied the fluorescence anisotropy of the inclusion complexes of 3HF in α-, β-, and γ-CDs compared to 3HF in the aqueous medium. The fluorescence anisotropy of these probes decreased following the order α-, β-, and γ-CD, which is attributed to the disruption of the 3HF-water network in an aqueous medium [46]. It can be stated that the micro-environment of 3HF derivatives was able to alter and prevent the self-aggregation effectively by using CDs, especially γ-CD. From the investigation on ESPT processes of 3HF in β-, and γ-CDs [45,47], the intensity of K* of 3HF/γ-CD inclusion complex is higher than that of 3HF/β-CD inclusion complex. Consequently, the encapsulation in CDs should be a possible method to tune the fluorescence emission of 3HF derivatives and other hydrophobic compounds [45,46,47,49,50,51,52].

From the studies of the multi-spectroscopic approaches and molecular docking of the encapsulation of 3HF in different small-ring CDs, the 3HF/γ-CD complex showed the strongest interaction, and it provided a higher fluorescent yield than that in water medium [45,46,47]. Understanding the role of CD in enhancement of the fluorescent yield at atomic level might help us to be able to adjust the fluorescent wavelength to fit for fluorescence probes for bio-labeling in aqueous medium. However, to the best of our knowledge, the detailed information of ESPT reaction in the S_0_ and S_1_ states of 3HF in γ-CD at atomic level has not been reported. In this work, we aimed to systematically investigate the effect of a water molecule on the photophysical properties and ESPT reactions of an isolated 3HF and its encapsulation. All-atom molecular dynamics (MD) simulations for 300 ns were performed to study the structure and dynamics properties of the two possible 3HF/γ-CD complexes. The detailed information of each complex both in the S_0_ and S_1_ states was then investigated using density functional theory (DFT) and time-dependent DFT (TD-DFT) methods. The important distances and simulated infrared (IR) vibrational spectra from the optimized structures as well as the topology analysis were used to describe the hydrogen-bonded strength. The frontier molecular orbitals (MOs) were analyzed to provide the charge distribution of the complex. The simulated absorption and emission spectra were calculated and compared with the experimental data. Moreover, the energies of E and K forms of 3HF in each system at the S_0_ and S_1_ states were discussed to explain why the fluorescent yield of K* in aqueous medium increases when encapsulating 3HF into γ-CD.

## 2. Results and Discussion

### 2.1. Possible Inclusion Complexes

**Figure 1:**
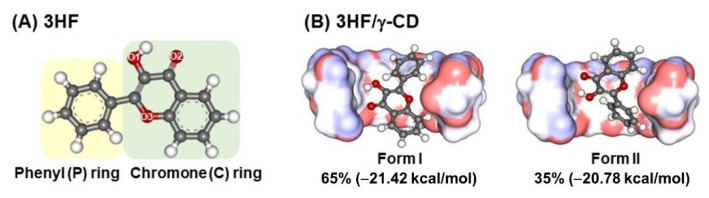
(**A**) Chemical structure of 3-Hydroxyflavone, 3HF. (**B**) Docked structures of the two possible 3HF/γ-CD complexes, Form I and Form II, where their percentage of occurrence and the lowest interaction energy retrieved from 100 independent docking runs are also given.

From the docking study, 3HF could form two possible inclusion complexes with γ-CD through its chromone ring (C-ring, Form I) or phenyl ring (P-ring, Form II) insertion into the hydrophobic cavity as depicted in Figure 1B. Although a higher occurrence (65%) was found for Form I, the interaction energies of both forms were likely comparable (Form I: −21.42 kcal/mol and Form II: −20.78 kcal/mol). Additionally, the previous docking studies [46,47] suggested that Form II was more stable. Thus, in the present work the 3HF/γ-CD complexes in both forms were further studied by MD simulations and DFT calculations.

### 2.2. 3HF Mobility in γ-CD Cavity and Water Accessibility

**Figure 2:**
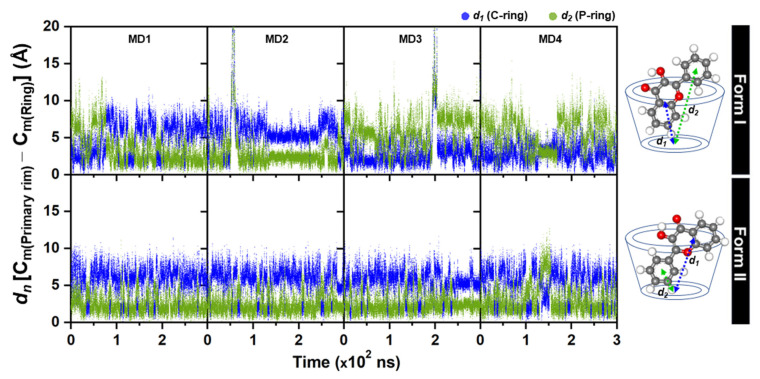
The plots of distance measured from the C_m_ of each 3HF ring to the C_m_ of the primary rim of γ-CD (all 7 O6 atoms) for the four MD simulations MD1-MD4 with different initial structures of complexes in Form I and Form II.

**Figure 3:**
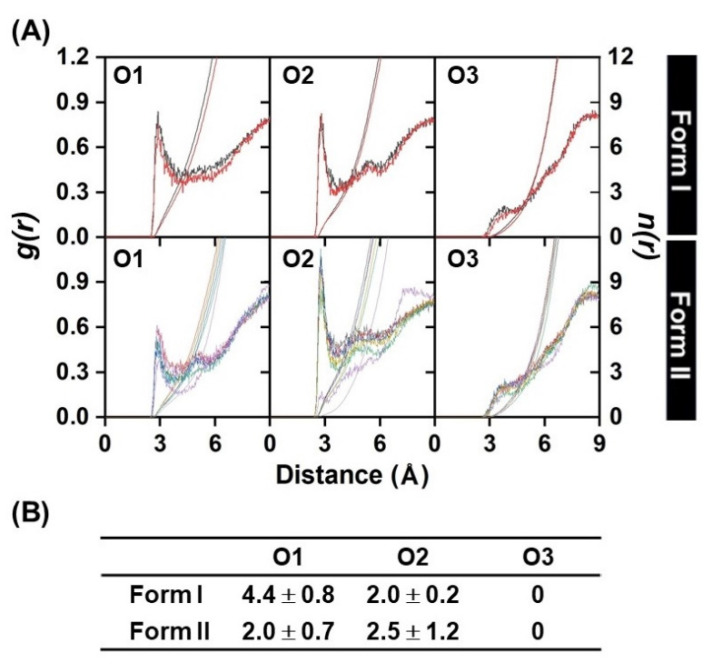
(**A**) Radial distribution function (RDF or *g*(*r*)) of water oxygen atoms around the O1-O3 atoms of 3HF encapsulated in the γ-CD cavity over the last 50-ns MD simulations for Form I (MD3-MD4) and Form II (MD1-MD4 with initial structures in this form and additional MD1-MD2 from re-encapsulation process found in Form I). (**B**) Average integration number, *n*(*r*), up to the first minimum derived from RDF plots, corresponding to the number of water molecules pointing toward the focused oxygens of 3HF.

To study the inclusion complexes in solution, the four different initial structures of Form I and Form II obtained from molecular docking and QM calculation were simulated by 300-ns MD simulations (MD1-MD4). All trajectories were analyzed and discussed as follows. The plots of RMSD and R_gyr_ of complex in Supplemental Figure S1 suggested that in Form I 3HF spontaneously released from the γ-CD cavity at ~68 ns, ~54 ns, and ~198 ns for MD1, MD2 and MD3. Interestingly, it feasibly moved back to form a complexation with γ-CD, resulting in Form I (MD3 ~201 ns) and Form II (MD1 ~70 ns and MD2 ~57 ns) as considerably seen by the plot of distance between the center of mass (C_m_) of each 3HF ring and the C_m_ of the primary rim of the γ-CD in Figure 2, and the plot of distance between the C_m_ of each 3HF ring and the C_m_ of the secondary rim of the γ-CD in Supplementary Materials Figure S2. This is in contrast for Form II, in which the 3HF was well encapsulated inside the hydrophobic cavity in all MD1-MD4 systems throughout the simulation time (RMSD of 1.2–4.0 Å, and R_gyr_ of 6.0−7.0 Å). Moreover, it can be noticeable that the both rings of 3HF in Form I (MD3 and MD4) were fluctuated higher than those in Form II, suggesting that the complex in Form II was found to be more stable in aqueous solution; in other words, the P-ring insertion is the suitable binding mode of 3HF for encapsulation with γ-CD in consistent with the lower water accessibility to the encapsulated 3HF (*n(r)* of 2.0 ± 0.7 and 2.5 ± 1.2 at O1 and O2, respectively, in Figure 3). In Form I, the hydroxyl oxygen O1 of 3HF positioned closer to the wider rim of γ-CD had a significantly higher interaction with waters (4.4 ± 0.8), and in vice versa less waters (2.0 ± 0.2) can access to the carbonyl oxygen O2. No peak detected within ~2.8 Å of the O3 of 3HF, suggesting that this atom had a very weak hydration interaction as found in some flavonoids/CD complexes [53,54]. It is worth noting that such accessible water molecules at O1 and O2 sites may involve into proton transfer processes either blocking ESIntraPT or assisting ESInterPT in 3HF/γ-CD inclusion complex. To study the water assisted PT in 3HF/γ-CD either ground state or excited state, the model of 3HF/γ-CD with a water molecule placed between these two sites of 3HF was further investigated by DFT and TD-DFT calculations and discussed in the next sections.

### 2.3. Structural Optimizations

**Figure 4:**
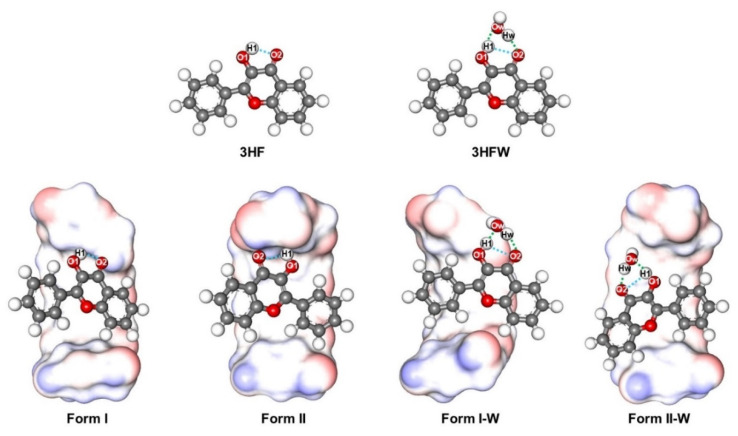
All S_0_ optimized structures of 3HF, 3HFW, and the different conformations of the 3HF/γ-CD inclusion complexes (Form I and Form II) as well as its inclusion complex with a water molecule (Form I-W and Form II-W) computed by PBE0/def2-SVP level of theory. The blue and green dot lines represent intraHB in 3HF and interHBs between 3HF and a water molecule, respectively.

All optimized E forms of 3HF, 3HFW, and 3HF encapsulated in the γ-CD cavity (Form I, Form II, Form I-W and Form II-W) with important labeled atoms and distances (O−H covalent bonds, intraHB, and interHBs) in the S_0_ state are shown in Figure 4, where the measured distances in the S_0_ and S_1_ states are summarized in Table 1.

From Table 1, for the compounds without a water molecule (3HF, Form I, and Form II), the O1–H1 covalent bond at the 3HF hydroxyl group slightly increases from the S_0_ to S_1_ states around 0.015–0.034 Å. Whereas, the length of O2⋯H1 intraHB between the hydroxyl group and carbonyl group of 3HF significantly decreases in the S_1_ state at 0.214, 0.174, and 0.097 Å for 3HF, Form I, and Form II, respectively. The length of O2⋯H1 intraHB of Form I and Form II is longer than that of the isolated 3HF because the O2 of 3HF forms a stronger interHB with a hydroxy group at the primary rim of γ-CD (O6–H) for Form I and at the secondary rim of γ-CD (O2–H) for Form II in the S_1_ state. Overall, these results indicate that the strength of an intraHB in the S_1_ state of 3HF, Form I, and Form II is stronger than that in the S_0_ state. Consequently, the ESIntraPT process might easily occur in the S_1_ state.

For the compounds with a water molecule (3HFW, Form I-W, and Form II-W), a water molecule forms interHBs with 3HF, therefore an O2⋯H1 intraHB of these complexes is dramatically longer than that of the compounds without a water molecule. It can be stated that a water molecule might block ESIntraPT process and support ESInterPT process. The O1–H1 and Ow–Hw covalent bonds of 3HFW/Form II-W are slightly increased from the S_0_ to S_1_ states at 0.069/0.074 Å for O1–H1 and 0.024/0.023 Å for Ow–Hw covalent bonds. While the lengths of Ow⋯H1 and O2⋯Hw interHBs of 3HFW/Form II-W are significantly decreased from the S_0_ to S_1_ states around 0.236/0.237 Å for Ow⋯H1 and 0.151/0.152 Å for O2⋯Hw interHBs, so ESInterPT may easily take place more than ESIntraPT for 3HFW and Form II-W due to the strong interHBs in the S_1_ state. For Form I-W, the length of Ow⋯H1 interHB decreases (0.082 Å), while the length of O2⋯Hw interHB increases (0.095 Å) in the S_1_ state because Hw of a water molecule forms a stronger interHB with O6 at a primary rim of γ-CD instead of O2 acceptor of 3HF in the S_1_ state. Consequently, ESInterPT of Form I-W is quite difficult to occur because the O2⋯Hw interHB between 3HF and a water molecule is weaker (elongated length) in the S_1_ state. Furthermore, the strength of intraHBs and interHBs in the S_1_ state will be further discussed in the next section.

### 2.4. O–H Stretching and Topology Analysis

In general, the strength of the intraHBs and interHBs in the S_1_ state could be revealed based on monitoring the red-shift of vibrational modes involving the hydrogen-bonded formation and the topology analysis at bond critical points (BCPs) using the QTAIM method. The calculated IR spectra of all studied compounds in the conjunct vibrational regions of the O–H stretching modes related with PT process both in the S_0_ and S_1_ states are listed in Table 2. These O–H stretching modes can be classified into the O1–H1 stretching mode of 3HF and Ow–Hw stretching mode of a water molecule.

For 3HF, the O1–H1 stretching vibrational mode of 3HF is located at 3544 cm^−1^ and 3003 cm^−1^ for the S_0_ and S_1_ states, respectively, giving a large red-shift of 541 cm^−1^. Moreover, the large red-shift of the O1–H1 stretching vibrational mode is also observed for Form I (367 cm^−1^) and Form II (244 cm^−1^). Therefore, the strength of the O2⋯H1 intraHB for these compounds is increased in the S_1_ state providing ESIntraPT process.

For the compounds with a water molecule, the O1–H1 stretching vibrational modes of 3HFW, Form I-W, and Form II-W are located around 2967–3129 cm^−1^ in the S_0_ state. Note that these vibrational modes changed to be around 2575–2599 cm^−1^ in the S_1_ state, which evidently demonstrates that the red-shift is induced by the strengthening of the O1–H1⋯Ow interHB after photoexcitation. Similarly, the Ow–Hw stretching modes of these compounds are also red-shifted. In addition, the red-shift value of 3HFW and Form II-W is larger than that of Form I-W, indicating that the O1–H1⋯Ow and Ow–Hw⋯O2 intermolecular hydrogen-bonded strength of 3HFW and Form II-W in the S_1_ state is stronger than that of Form I-W. Overall, these results show that O–H stretching vibrational frequencies shift to lower frequencies in the S_1_ state compared with the S_0_ state, which confirms that the hydrogen bonding interaction is stronger in the S_1_ state.

A topology analysis of the electron density was used to further determine the strength of the intraHB and interHBs in the excited-state structures (E* form) of all compounds. The following parameters at BCPs were analyzed: the electron density *ρ*(r), the potential energy density V(r), the Laplacian of the electron density ∇^2^*ρ*(r), the Lagrangian kinetic energy G(r), the Hamiltonian kinetic energy density H(r), and the electron delocalization index (DI) between the proton acceptor and transferred proton, which are an O2⋯H1 intraHB, and the Ow⋯H1 and O2⋯Hw interHBs for all studied compounds. Additionally, the hydrogen-bonded energy (E_HB_) can be calculated by using the Espinosa’s equation: $E_{HB}=\frac{1}{2}\left|V(r_{BCP})\right|$. These results are summarized in Table S1 in the Supplementary Materials. From Table S1, E_HB_ of O2⋯H1 intraHB of 3HF (0.0224 a.u.) is the highest among the compounds without a water molecule. Then, E_HB_ is slightly decreased for Form I (0.0175 a.u.) and Form II (0.0111 a.u.). These results indicate that the intraHB of isolated 3HF is stronger than that the inclusion complexes. Nevertheless, this intraHB of all compounds can facilitate ESIntraPT processes. For the compounds with a water molecule, E_HB_ of Ow⋯H1 and O2⋯Hw of 3HFW/Form II-W are 0.0696/0.0649 and 0.0306/0.0317 a.u., respectively. The Ow⋯H1 interHB of 3HFW/Form II-W is stronger than the O2⋯Hw interHB, indicating that the proton might transfer via a water molecule from O1–H1 bond-breaking before Ow–Hw bond-breaking. However, only E_HB_ of Ow⋯H1 of Form I-W is obviously dropped (0.0253 a.u.). The result implies that the ESInterPT process might occur in 3HFW/Form II-W better than Form I-W. Overall, it can be observed that the intraHB and interHBs are strengthened in the S_1_ state confirmed by the shorter distances of important bonds involving in the ESPT process, the red-shift observed by IR vibrational spectral calculations, and a high value of E_HB_ from topology analysis of the electron density.

### 2.5. Frontier MOs Analysis and Simulated Spectra

**Figure 5:**
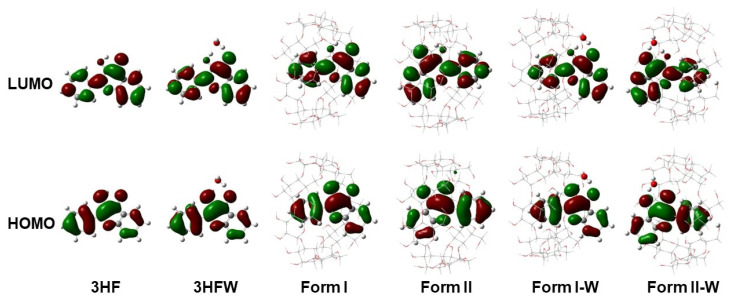
Frontier MOs of all studied compounds.

To further investigate behaviors of charge distribution and charge transfer in the S_1_ state, the frontier MOs of the highest occupied molecular orbitals (HOMO) and the lowest unoccupied molecular orbitals (LUMO) of all studied compounds were analyzed because the main electronic transition is only related with these orbitals in range of 98% (HOMO → LUMO), which is assigned as π to π* characters, and illustrated in Figure 5. It can be noted that electron density of both HOMO and LUMO is fully localized on the 3HF moieties and no electron density is located on water or γ-CD, indicating that no intramolecular charge transfer within 3HF and no intermolecular charge transfer between 3HF and water or γ-CD. Moreover, the HOMO and LUMO are localized on different parts of 3HF. For the HOMO orbitals, the electron density is distributed more on P-ring and partially on C-ring of 3HF. Whereas, that of LUMO is distributed completely on the whole molecule of 3HF.

The UV-Vis absorption and emission spectra of all studied compounds were simulated based on their optimized S_0_ and S_1_ structures, respectively and then plotted in Figure S3. Furthermore, the absorption band maxima of E form (λ_abs of E_), emission band maxima of E* (λ_emis of E*_) and K* forms (λ_emis of K*_), the excitation energy (E_ex_), and the oscillator strength (*f*) as well as the major MOs contribution (%) of the absorption band for all compounds are reported in Table 3. From the detailed information in Table 3, the simulated absorption peaks of the complexes without and with a water molecule are around 341–345 nm and 350–353 nm, respectively, which are in good agreement with the experimental value of 341 nm for 3HF in water and 340 nm for 3HF in γ-CD [46,47]. Moreover, the predicted maximum wavelength for dual emission spectra of all studied compounds is also consistent with the experimental data [46,47], in which the λ_emis of E*_ in water and in γ-CD are reported at 410 and 404 nm, respectively, while the λ_emis of K*_ in water and in γ-CD are 511 and 538 nm. The deviations from the experimental data around 59–69 nm (0.52–0.61 eV) indicate that the chosen method at TD-PBE0/def2-SVP level of theory is adequate to describe the electronic spectra and provide the insight understanding of the ESPT process.

The relative energy of E and K forms of all studied complexes at the S_0_ and S_1_ states was investigated to explain the ESPT phenomena as illustrated in Table 4. The results show that the E form is more stable than the K form in the S_0_ state for all complexes with the energy differences at 6.63–16.85 kcal/mol. Moreover, most of K forms were stabilized in the γ-CD cavity except only Form I. However, in the S_1_ state, the K* form is more stable than the E* form for all complexes. It is predicted that both ESIntraPT and ESInterPT processes are favorable in the S_1_ state but not in the S_0_ state. In case of the complexes without a water molecule (Form I and Form II), K* of Form II is more stable than Form I. So, the ESIntraPT process of Form II may be more effective than that of Form I, related to the MD results that Form II is favorably more stable. For the complexes with a water molecule, the ESInterPT via interHBs network is feasible to occur in both Form I-W and Form II-W especially Form II-W because of the slightly higher oscillator strength and the lower energy of K*. In addition, from our previous work, the ESInterPT of 3HFW is hard to occur due to the higher ESInterPT barrier and the rearrangement of a water molecule surrounding 3HF [40]. Therefore, it can be predicted that the encapsulating 3HF into γ-CD assists the disruption of the 3HF-water network in aqueous solution leading to an increment of the fluorescent yield of K* in aqueous solution from the ESIntraPT process.

## 3. Methodology

### 3.1. Model Preparation

The 3D structure of 3HF (Figure 1A) was generated by GaussView version 6.0 [55]. To investigate the effect of water on PT process, a water molecule near the PT site of 3HF was placed between the H1 and O2 atoms to model 3HF with a water molecule (3HFW). The 3HF and 3HFW structures were fully optimized with DFT and TD-DFT methods using Gaussian 16, Revision C.01 [56]. The γ-CD structure extracted from the co-crystal structure of cyclo/maltodextrin-binding protein in complex with γ-CD (PDB code: 2ZYK) was used in this study. To obtain the inclusion complex, encapsulation of the optimized 3HF into γ-CD’s cavity was performed by molecular docking with 100 runs using the CDOCKER module in the Accelrys Discovery Studio 2.5 (Accelrys Software Inc., San Diego, CA, USA). The three docked inclusion complexes with the lowest CDOCKER interaction energy for the chromone ring (C-ring, Form I) and phenyl ring (P-ring, Form II) insertion into the hydrophobic cavity of the γ-CD were selected for MD simulations (MD1-MD3 for Form I and Form II). The most stable 3HF/γ-CD inclusion complexes in Form I and Form II from a docking study were fully optimized at PBE0/def2-SVP level of theory for investigating PT reaction. Note that, these Form I and Form II were used as the additional initial structures for MD simulations, MD4. To study the effect of water associated in PT for 3HF encapsulated in γ-CD, a water molecule was added to form interHBs with 3HF both in Form I and Form II namely Form I-W and Form II-W, respectively. Then, Form I-W and Form II-W were fully optimized with DFT and TD-DFT methods.

### 3.2. Molecular Dynamics Simulations

MD simulations of the 3HF/γ-CD complexes in Form I and Form II using the four different initial structures (eight complexes in total) were carried out using the AMBER16 program package [57]. The partial atomic charges and parameters of 3HF were generated in accordance with the previous standard procedures [58,59,60]. The general AMBER force field [61] and the Glycam06j carbohydrate force field [62] were applied for 3HF and γ-CD, respectively. The models were solvated by a truncated octahedral box of TIP3P water molecules with a spacing distance of 15 Å from complex. Afterward, all added water molecules were minimized using 1000 steps of steepest descent (SD) and continued by 3000 steps of conjugated gradient (CG). Next, the minimizations with the same steps were performed on the whole system. All studied models were heated up from 10 K to 298 K with a constant volume ensemble (NVT) for 100 ps and followed by all-atom MD simulations with a constant pressure ensemble (NPT) at 1 atm and 298 K for 300 ns. All chemical bonds involving hydrogen were constrained using the SHAKE algorithm [63]. The particle mesh Ewald’s method [64] was performed for the treatment of the long-range interactions. The cpptraj module of AMBER16 program was used to calculate the root-mean-square displacement (RMSD), the radius of gyration (R_gyr_), and the distance between guest and host molecules as well as the radial distribution function (RDF).

### 3.3. Quantum Chemical Calculations

All E and K forms of 3HF, 3HFW, and 3HF/γ-CD inclusion complexes in Form I and Form II in the S_0_ and S_1_ states were studied by PBE0 and TD-PBE0 methods, respectively with def2-SVP basis set by using Gaussian 16, Revision C.01 [56]. The solvation effect was taken into account by means of the non-equilibrium implementation of the conductor polarized continuum model (C-PCM) framework [65,66], so-called PCM-LR [67]. To confirm that the optimized structures (E, K, E*, and K*) are located at the local minimum, no imaginary frequency from vibrational calculations was found for all optimized structures both in S_0_ and S_1_ states. A hydrogen-bonded strength was determined by the important distance parameters involving ESIntraPT and ESInterPT processes namely the covalent O–H bond of 3HF and a water molecule, the intraHB between proton donor and proton acceptor of 3HF, and the interHBs between 3HF and a water molecule for the case study of water assisted effect. The strength of all intraHB and interHBs was further confirmed by the red-shift values of the O–H stretching vibrational frequencies between the S_0_ and S_1_ states from the simulated IR spectra, together with the topology analysis at bond critical point (BCP) from quantum theory of atoms in molecules (QTAIM) performed by Multiwfn [68], which was employed in previous studies [69,70,71]. The electronic spectra and frontier MOs were also calculated. Additionally, the absorption and emission spectra of all complexes were simulated to investigate the photophysical properties.

## 4. Conclusions

The effect of water microsolvation on the strength of hydrogen bonding, ESIntraPT and ESInterPT reactions as well as photophysical behaviors of 3HF and its inclusion complexes in γ-CD has been systematically studied using MD simulations and DFT/TD-DFT at PBE0/TD-PBE0 with def2-SVP basis set. Two possible 3HF/γ-CD inclusion complexes; C-ring and P-ring insertions (Form I and Form II), were observed from molecular docking. From MD results, a lower 3HF mobility in the hydrophobic cavity and a lower water accessibility to the encapsulated 3HF in Form II suggest that this form is favorably more stable. From the static calculation results, the strength of hydrogen bonding of all studied compounds increases upon photoexcitation into the S_1_ state leading to being easier deprotonation, confirmed by the change of important bond lengths (the increasing of the covalent O–H bond length of proton donor, together with decreasing of the O⋯H intraHBs and interHBs), the red shift of the O–H stretching modes, and the bond energy from the topology analysis. In addition, frontier MOs confirm that the main contribution for vertical S_0_ to S_1_ transition is π to π* attributed from HOMO (π) to LUMO (π*). For simulated spectra, the λ_abs of E_, the λ_emis of E*_ and the λ_emis of K*_ are in good agreement with the experimental data (in the range of 0.52–0.61 eV relative differences) indicating that the present method is adequate to provide the information on their spectra and the possibility of ESPT processes. Besides, the ESIntraPT processes of the inclusion complexes (Form I and Form II) can occur easily like in the case of 3HF in aprotic solvents. Furthermore, the ESInterPT processes via interHBs of Form I-W and Form II-W inclusion complexes are feasible to take place. In addition, K* of Form II/Form II-W is more stable than that of Form I/Form I-W due to the lower energy and the higher oscillator strength. Consequently, the ESIntraPT and ESInterPT might be likely to occur in P-ring insertion in accordance with the MD results, in which P-ring insertion is the majority of the 3HF/γ-CD inclusion complexes with lower water accessibility. However, it is already known that the ESInterPT of 3HF in aqueous solution is hard to occur due to the higher ESInterPT barrier and the higher fluctuation of the water-rearrangement surrounding 3HF, which leads to a decrease of the fluorescence intensity. Thus, from the present work, we found that 3HF is stable inside the γ-CD hydrophobic cavity and promotes ESIntraPT by suppressing the 3HF-water network. This leads to the increment of fluorescent intensity. In the other word, the fluorescence intensity of K* could be efficiently tuned via host-guest complexation.

## Figures and Tables

**Table 1:** A summary of the important bonds and distances involving PT process of enol form for all complexes in the S_0_ and S_1_ states.

Compound	Important Bond Distance (Å)									
	S_0_ State					S_1_ State				
	O1–H1	O2⋯H1	Ow⋯H1	Ow–Hw	O2⋯Hw	O1–H1	O2⋯H1	Ow⋯H1	Ow–Hw	O2⋯Hw
**3HF**	0.983	1.920				1.017	1.706			
**3HFW**	1.000	2.387	1.630	0.984	1.723	1.069	2.439	1.394	1.008	1.572
**Form I**	0.979	1.978				1.002	1.804			
**Form II**	0.981	2.177				0.996	2.080			
**Form I-W**	1.009	2.390	1.585	0.977	1.808	1.030	2.415	1.503	0.978	1.903
**Form II-W**	1.004	2.429	1.612	0.983	1.733	1.078	2.452	1.375	1.006	1.581

**Table 2:** A summary of the values of the O1–H1 and the Ow–Hw stretching vibrational modes of enol form for all compounds in both S_0_ and S_1_ states and their spectral shifts (Δυ in cm^−1^).

Compound	Wavenumber (cm^−1^)					
	O1–H1			Ow–Hw		
	S_0_	S_1_	Δυ	S_0_	S_1_	Δυ
**3HF**	3544	3002	541			
**3HFW**	3129	2599	530	3510	3074	436
**Form I**	3617	3250	367			
**Form II**	3574	3330	244			
**Form I-W**	2967	2575	392	3662	3499	163
**Form II-W**	3063	2577	486	3526	3111	415

**Table 3:** UV/Vis absorption band maxima of enol form (λ_abs of E_), emission band maxima of enol (λ_emis of E_) and emission band maxima of keto forms (λ_emis of K_), the excitation energy (E_ex_), and the oscillator strength (*f*) as well as their major contribution (%) calculated by TD-PBE0/def2-SVP level of theory.

Compounds	UV/Vis Absorption				Emission					
	λ_abs of E_ (nm)	E_ex_ (eV)	*f*	MOs (%contribution)	λ_emis of E*_ (nm)	E_ex_ (eV)	*f*	λ_emis of K* _ (nm)	E_ex_ (eV)	*f*
**3HF**	341	3.63	0.4907	HOMO→LUMO (98%)	390	3.18	0.5454	514	2.41	0.4632
**3HFW**	351	3.53	0.4857	HOMO→LUMO (98%)	408	3.04	0.5335	504	2.46	0.4619
**Form I**	345	3.59	0.4618	HOMO→LUMO (98%)	392	3.16	0.5123	524	2.36	0.3692
**Form II**	345	3.59	0.4081	HOMO→LUMO (98%)	393	3.15	0.4689	528	2.35	0.3792
**Form I-W**	353	3.51	0.4288	HOMO→LUMO (98%)	399	3.11	0.5213	529	2.34	0.3708
**Form II-W**	350	3.54	0.4174	HOMO→LUMO (98%)	412	3.01	0.4575	508	2.44	0.3899

**Table 4:** The relative energy and computed energy differences between the E and K forms (Δ*E* = *E*_enol −_ *E*_keto_) in the S_0_ and S_1_ states for all complexes.

Complex	Relative Energy (kcal/mol)					
	S_0_		S_1_		Δ*E* at S_0_	Δ*E* at S_1_
	E	K	E*	K*		
**3HF**	0	10.74	75.19	65.81	10.74	−9.38
**3HFW**	0	10.27	74.92	63.41	10.27	−11.51
**Form I**	0	16.85	74.79	71.16	16.85	−3.63
**Form II**	0	8.47	72.39	64.68	8.47	−7.71
**Form I-W**	0	6.63	71.24	63.43	6.63	−7.81
**Form II-W**	0	8.35	73.12	64.72	8.35	−8.40

## Supplementary Materials

The following are available online. Figure S1: RMSD plots for all atoms and R_gyr_ of the two orientations of C-ring insertion (Form I), and P-ring insertion (Form II) inclusion complexes for four different MD runs of each system, Figure S2: The plots of distance measured from the C_m_ of each 3HF ring to the C_m_ of the secondary rim of γ-CD (all 7 O2 atoms) for the four MD simulations MD1-MD4 with different initial structures of complexes in Form I and Form II, Figure S3: The simulated absorption spectra of E, and the simulated emission spectra of E* and K* for all studied compounds computed at TD-PBE0/def2-SVP level of theory, Table S1: Electron density ρ(r), the Lagrangian kinetic energy G(r), potential energy density V(r), the Hamiltonian kinetic energy density H(r), the Laplacian of the electron density ∇^2^ρ(r), the electron delocalization index (DI), and hydrogen-bonded energy (E_HB_) at selected BCPs in the S_1_ state (a.u.) for all compounds.

## Sample Availability

Samples of the compounds are not available from the authors.
