# Supplementary material for: Effect of Water Microsolvation on the Excited-State Proton Transfer of 3-Hydroxyflavone Enclosed in γ-Cyclodextrin
Source: Molecules. 2021 Feb 5;26(4):843. doi: 10.3390/molecules26040843 (PMC7914428; doi:10.3390/molecules26040843)
Supplement: Supplementary file 1 [file molecules-26-00843-s001.pdf]

molecules

Supplementary Materials

Effect of Water Microsolvation on Excited-State Proton Transfer of 3-Hydroxyflavone Enclosed in γ-Cyclodextrin

Khanittha Kerdpol [1], Rathawat Daengngern [2,3], Chanchai Sattayanon [4], Supawadee Namuangruk [4], Thanyada Rungrotmongkol [5,6,7], Peter Wolschann [8,9], Nawee Kungwan [10,11,*] and Supot Hannongbua [1,7,*]

1 Center of Excellence in Computational Chemistry (CECC), Department of Chemistry, Faculty of Science, Chulalongkorn University, Bangkok 10330, Thailand
2 Department of Chemistry, Faculty of Science, King Mongkut's Institute of Technology Ladkrabang, Bangkok 10520, Thailand
3 Integrated Applied Chemistry Research Unit, Faculty of Science, King Mongkut's Institute of Technology Ladkrabang, Bangkok 10520, Thailand
4 National Nanotechnology Center (NANOTEC), NSTDA, 111 Thailand Science Park, Pahonyothin Road, Klong Luang, Pathum Thani 12120, Thailand
5 Structural and Computational Biology Research Unit, Department of Biochemistry, Faculty of Science, Chulalongkorn University, Bangkok 10330, Thailand
6 Program in Bioinformatics and Computational Biology, Graduate School, Chulalongkorn University, Bangkok 10330, Thailand
7 Molecular Sensory Science Center, Faculty of Science, Chulalongkorn University, Bangkok 10330, Thailand
8 Department of Pharmaceutical Chemistry, University of Vienna, 1090 Vienna, Austria
9 Institute of Theoretical Chemistry, University of Vienna, 1090 Vienna, Austria
10 Department of Chemistry, Faculty of Science, Chiang Mai University, Chiang Mai 50200, Thailand
11 Center of Excellence in Materials Science and Technology, Chiang Mai University, Chiang Mai 50200, Thailand
* Correspondence: naweekung@gmail.com (N.K.); supot.h@chula.ac.th (S.H.); Fax: +66-53-892277 (N.K.); Fax: +66-22-187598 (S.H.)

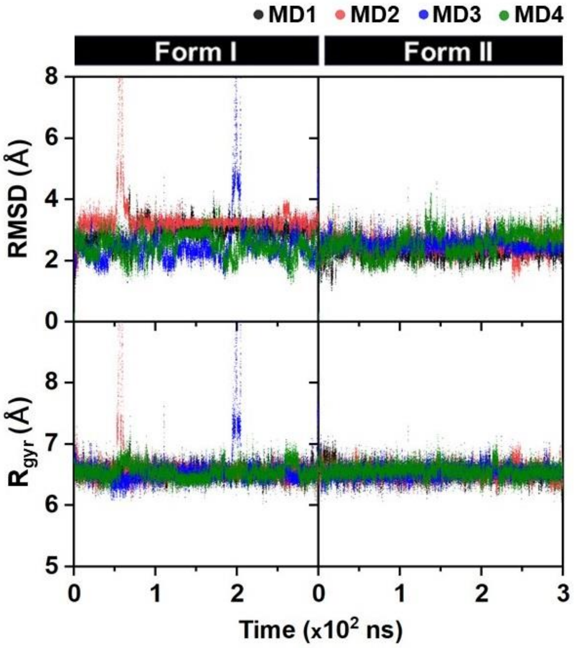

Figure S1. RMSD plots for all atoms and R_{gyr} of the two orientations of C-ring insertion (Form I), and P-ring insertion (Form II) inclusion complexes for four different MD runs of each system.

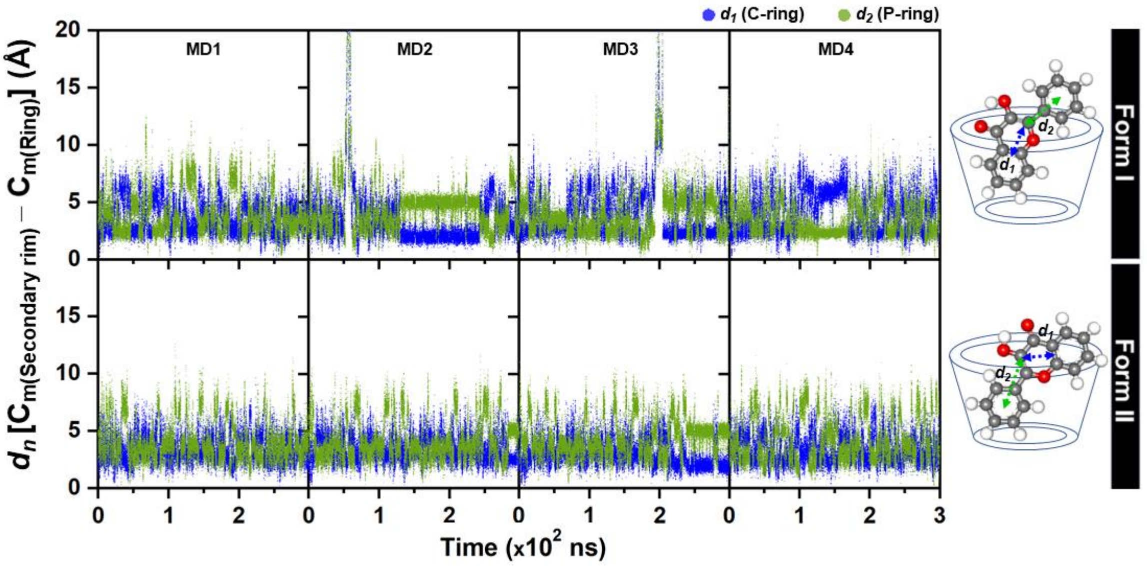

Figure S2. The plots of distance measured from the C_m of each 3HF ring to the C_m of the secondary rim of γ-CD (all 7 O2 atoms) for the four MD simulations MD1-MD4 with different initial structures of complexes in Form I and Form II.

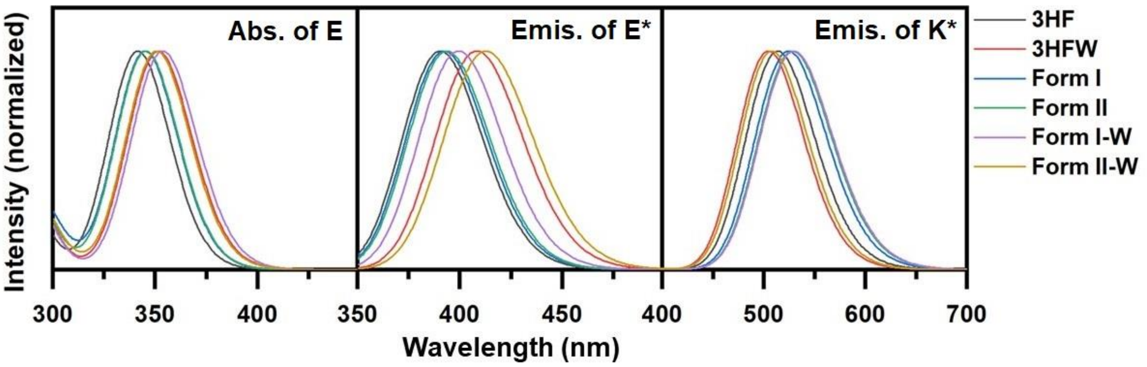

Figure S3. The simulated absorption spectra of E, and the simulated emission spectra of E* and K* for all studied compounds computed at TD-PBE0/def2-SVP level of theory.

Table S1. Electron density ρ(r), the Lagrangian kinetic energy G(r), potential energy density V(r), the Hamiltonian kinetic energy density H(r), the Laplacian of the electron density $\nabla^2\rho$(r), the electron delocalization index (DI), and hydrogen-bonded energy (E_{HB}) at selected BCPs in the S_1 state (a.u.) for all compounds.

Cluster	BCPs	ρ(r)	G(r)	V(r)	H(r)	$\nabla^2\rho$(r)	DI	E_{HB}
**3HF**	**O2···H1**	0.0516	0.0418	-0.0448	-0.0031	0.1549	0.6756	0.0224
**3HFW**	**Ow···H1**	0.1007	0.0764	-0.1298	-0.0534	0.0918	0.0054	0.0649
	**O2···Hw**	0.0633	0.0538	-0.0634	-0.0096	0.1769	0.1481	0.0317
**Form I**	**O2···H1**	0.0414	0.0342	-0.0350	-0.0009	0.1330	0.0826	0.0175
**Form II**	**O2···H1**	0.0249	0.0218	-0.0221	-0.0003	0.0860	0.0346	0.0111
**Form I-W**	**Ow···H1**	0.0575	0.0435	-0.0505	-0.0070	0.1461	0.0000	0.0253
	**O2···Hw**	0.0636	0.0553	-0.0646	-0.0093	0.1837	0.0159	0.0323
**Form II-W**	**Ow···H1**	0.1052	0.0785	-0.1391	-0.0606	0.0717	0.0077	0.0696
	**O2···Hw**	0.0618	0.0528	-0.0612	-0.0084	0.1774	0.0000	0.0306
